# Central Dental Association of Northern New Jersey

**Published:** 1892-05

**Authors:** 


### CENTRAL DENTAL ASSOCIATION OF NORTHERN NEW JERSEY.

Regular meeting, Monday evening, October 19, 1891, in the dining-parlors of S. & J. Davis, Broad Street, Newark. The President, Dr. C. W. F. Holbrook, in the chair.

The roll was called by the secretary.

On motion of Dr. Levy, the regular order of business and the reading of the minutes was dispensed with until after the reading of the papers.

T. Y. Sutphen, M.D., then considered the subject he desired to bring before the Association, that of "The Use of the Eyes in Dentistry."

By the use of a model the construction of a normal eye was explained; how the muscle of accommodation altered the convexity of the lens by its contraction; and, stating the fact that the eye was comparatively at rest when adapted for long distance, and that the greatest muscular effort was found in focussing upon near objects, he gave a comprehensive explanation of far-sightedness and near-sightedness; that the former was due to an eye being too short, and allowing only distinct vision by a considerable effort, and so compensating for the shortened antero-posterior focal length; and the latter due to the eyeball being too long, thus bringing the focal point anterior to the surface of the retina, the perceptive membrane. He explained the correction of these errors by suitable glasses,—near-sightedness requiring a concave lens to scatter the rays of light, carrying the focal point farther back; while just the opposite glass, a convex one, was required in a far-sighted eye to bring the rays of light to a focus quicker, and in this way overcoming the short antero-posterior diameter, at the same time relieving the muscle of accommodation. The doctor referred to the error of refraction called astigmatism, and showed how this was due, not so much to any error in the length of the eyeball as to the scattering of a portion of the rays of light, while the greater proportion focussed properly. He spoke of the advantage of a near-sighted eye over a normal one, enabling the man who might be moderately near-sighted to do his work close by without glasses long after the age of forty-five. The action of the muscles of motion was described, and their importance was enlarged upon as a factor in bringing about astigmatic errors. Attention was directed to the influence an abnormal eye would have in giving a dentist discomfort, and advised the use of suitable corrective glasses where errors of refraction existed. Caution was given in the matter of overwork and too long hours, and advised frequent rest by leaving close work for a few moments. It was the opinion of the speaker that a north light was the best, and east light came next in value; and he suggested that the dentist should do as the oculist does,—bring his operating-chair up close to a window with the hangings removed, and where a good, even skylight might fall directly upon the point of operation. The doctor brought up the point of the value of artificial light in dental work, and thought it might be advisable to employ such illumination, providing the natural light was insufficient.

*The President.*—Gentlemen, this interesting subject is now open for discussion.

*Dr. Sutphen.*—I would like to have some of the gentlemen who have used artificial light express their opinion as to whether it is at all practical in dental surgery.

*Dr. Ottolengui.*—Mr. President, I was a little surprised that the essayist should have asked that question, because it was the one that I had in my mind to ask him to tell us about. A friend of mine bought a stomatoscope, and he told me he was going to make about double as much money in a year with it as he was making before, because he could work at night and on dark days, and later in the winter-time when the days are short. He had the stomatoscope about four months, and used it a great deal. I had an appointment with him one night which he did not keep, and the next day I received a note from him that he was very ill. I found him in bed, with an oculist attending him, and he was there about six weeks. The doctor ordered him to stop the use of the stomatoscope. In the mean time he had induced a friend to buy the instrument. He had a different kind; he bought it in England, and I suppose for that reason it was better. After having it about three months, he began to be troubled at night with insomnia, caused by constant pain in his head, which would occur after he had been asleep a few hours. He went from physician to physician, until some one told him to go to an oculist; and that oculist cured him with glasses, and the abandonment of the stomatoscope. That is the experience of two friends of mine with this instrument; and, while I regret their experience, I am very glad they had it before I bought mine, as I was just then on the point of doing.

*Dr. Sanger.*—Mr. President, I did own a stomatoscope; I do not own it now. As a part of my experience, I had to consult an oculist, and now I wear glasses. Whether the stomatoscope had anything to do with that or not, I do not know; but I think that the experience of other gentlemen who have tried to use artificial light will bear me out in the statement that no honest workman can do himself justice under it. A man who places himself under that unnatural strain, and risks his physical health for the small stipend that may be added to his income, shortens the days of usefulness for his eyes, if not the days of his own life. Therefore, I am utterly and entirely opposed to artificial light; and the brighter the light the more dangerous it is, because of the deep shadows which naturally follow.

I was much interested in the doctor's remarks, and I wish they had gone further for my instruction in the use and abuse of the eyes. It throws out a hint in regard to our attitude towards our patients. We are in the habit of standing beside the patient; yet I believe that it is good practice to place ourselves, as frequently as possible, nearly in front, because I believe we can do better work in that position. The use of the mirror in operations in the mouth I regard as bad, and should be avoided as much as possible. I think I have heard men boast that they could work with equal facility by looking into a mirror. I cannot, and what is more, I do not propose to try to learn how to do it, because I feel that in learning I would injure my eyes.

I think the doctor's hint is a valuable one, that artificial light should not be used, and for one I wish to emphasize it.

*Dr. Sutphen.*—My remarks concerning the use of artificial light by the dentist are from the stand-point of an oculist. We are almost constantly using artificial light, and I know of no unfortunate experience in the use of it. I can see how much more constant and trying must be the dentist's use of the eyes than in our work upon the eye or ear. It seems to me that the trouble which dentists find must not be so much in the use of artificial light as in the long-continued application under these conditions. The continued effort to see distinctly brings out the presence of refraction errors.

*Dr. Osmun.*—One point has not been touched upon. The oral cavity being partly closed, and the hand being used in holding the instrument, shows but a partial light at one side or the other, and there is constantly a shadow from the hand and the instrument; and I think that is one reason why artificial light should not be used in dental operations.

*Dr. Stockton.*—It is very true, that light, or rather sight, has as much to do with good dental operations as the dexterity of the hand; therefore it is very important that we take care of it, that we guard it in all the ways we can. I don't know whether Dr. Sutphen has ever tried the use of bent glasses for modifying the light. I was very sceptical about it. A gentleman told me that just as much light would come through an opaque glass as one entirely clear. I have put in my operating-room that kind of a window, and I have had a great deal more satisfaction in operating since than I ever had before.

*Dr. Ottolengui.*—Will you explain that bent glass?

*Dr. Stockton.*—It is a rough glass, such as is used in conservatory windows. The sun may shine almost directly on it, and yet not affect the eyes. If any of you are contemplating building your residence to suit yourselves, you should put in the windows such glass as I have put in mine, and you will have no trouble with your eyes.

I had an experience with artificial light a short time ago. It was simply two globes filled with water, with a gas-jet between them and a reflector. The reflector passes the light through these balls of water, which, of course, they magnify somewhat.

*Dr. Watkins.*—Mr. President, the glass that the doctor has referred to is simply the glass as it is cast, without being polished.

*Dr. Kingsley.*—Mr. President, I thought that an oculist should know a great deal, and I came over here for the purpose of hearing it. It is supposed, generally, that the men who are engaged in a particular profession are familiar with the subject; an oculist should know all about the eyes, about the use and management of light for the benefit of the eyes; not the management of light so that it will bring the patient into the hands of the oculist and bring him a fee; but the management of light so that it will keep the patient out of his hands and keep his eyes good. I do not know but that our friend can give us some information on that.

A couple of years ago I built an office, and the question of light was a serious matter with me in this way: that while for more than a quarter of a century before, I had worked with a northern light, I was now obliged by the circumstances of the locality, which I could not control, to adopt a southern light; and the question in my mind was whether I was going to be better or worse off on that account. I consulted with some of my confrères, and I found that some of them who had used a south light all their professional days would not have any other, and those who had used a north light, and then had come to use a south light, preferred the southern light, saying simply that they thought it was more healthy to have the sun in your room. Now, before that period, I had worked with a southern light, and had been disturbed by having the light shut off frequently by passing clouds. In speaking of my own experience with the north light, with those who had continued the use of the south, I was told that it was a trifling matter that was hardly to be considered in connection with the grand advantages of a south light; so I was obliged to adopt it, and accepted the situation without further inquiry, and I have not had the disturbance with this light that I feared I might have. But this is what I wish to know: how much light do I want, or need? Do I want a large window, letting in all that can come from the sky, or do I want a little light thrown upon my work, as I have seen in a gentleman's office, so managed as to bring the light from the sky and throw it horizontally to the operating chair? I found that some who were boasting very much over their good light had windows cut up with heavy bars, that it seemed to me would cast shadows; or having a good-sized window with only one or two panes of glass in that window. So I have never come to any definite conclusion or satisfaction in my mind as to what is the better light, the kind, its position, and its quality.

I was particularly interested this summer when in Berlin. I was in the office of one of our most noted practitioners, and he had a small window,—that is, he had a portion of the window filled up with some panes of stained glass; then the paper on the walls and the adornments of his room were very dark, with heavy hangings, and the room was filled with bric-a-brac and dark objects; yet it was his operating-room. When he dismissed the patient and gave up the rest of the day to me, I said to him, "How about your light here? Do you have all the light you want?" "Oh, yes," he said; "an abundance. You see I have this light concentrated. I don't have a great flood of light in my room, but just a small amount, and it comes right down exactly where I want it."

Now, in another office the dentist informed me he could not bear such a thing as that, because the dark objects absorbed the light. He wanted light paper, light walls, everything light about him. So, although I have been forty years practising dentistry, I don't know much about this branch of the subject, and it seems to me that our oculist ought to tell us something about the true science of the thing,—whether we want dark rooms and a concentrated light, or whether a light room and all the light we can get into them; whether we would see better out of doors, or at the extreme end of a contracted aperture.

*Dr. Faught.*—The particular point that leads me to speak to-night is a remark of the gentleman in reference to the use of the microscope. I have had some experience in that, and I think that one of the things which microscopists soon learn is that, while we want illumination, there is also a possibility of obtaining too much light. We learn to avoid bright illumination except in those cases where a particular negative is used, and to use neutral tints and a controlled light. I think the experience gained there is probably applicable to the use of light by dentists. I believe there is a possibility of getting more than is actually needed. We require thorough illumination, but more than that I believe to be injurious to the eye, and less than that equally injurious.

The gentleman who opened the subject stated that a north light was the best, and that the next best was an east light. I would like to have him inform us why he considers an eastern light next in value to a northern light.

*Dr. Sutphen.*—I think it is a generally-accepted fact that a north light is the best, because the direct sunlight does not reach the operating-room from that direction; the east light must come next in turn, on account of the time being shorter for direct rays from the sun during a dentist's working-hours.

*Dr. Watkins.*—Mr. President, I think that, as a rule, the dentists, unlike the physicians, get to work before the sun is high in the heavens; and therefore for two or three hours in the morning the east light would be very trying to work with. I consider this the poorest light, for the reason that in the morning it is very glaring. It is more trying to my eyes than a south light in the middle of the day. I think that from the north is preferable for dentists, because it is more even. This is at all times in the day just about the same. If we could have a constant south light, without clouds to obscure it, then I think I should prefer it, on account of its intensity. I like a very strong light. I have in my office a direct north light and a direct south light, and a skylight in each case. I think I have as fine a light as there is in America. I really think that a north light is the easiest on the eye; and, if I were going to build another office, I think I would have my principal office facing the north, with a skylight. Then you avoid having to depend upon curtains to regulate the rays, as you do with the south light. By having a set of curtains of different widths, they can be so arranged as to have the light come exactly where you want it at any time.

*Dr. Stockton.*—Mr. President, I would be glad to have Dr. Sutphen answer the pertinent question of Dr. Kingsley.

*Dr. Sutphen.*—I presume Dr. Kingsley meant to be complimentary when he called me an expert, but I really could not answer his question more definitely than in my remarks. I think it is a question to be answered by the personal experience of dentists themselves. As oculists, we do not strive to get a diffused light, but rather an even light from the sky, bringing our patients where a good strong skylight pours upon the point of operation.

Dr. Kingsley's statement that he had found so many men who used very different forms of light, and who were all satisfied with what they were using, was interesting but not at all satisfying to an inquiring mind. It would rather tend to prove that the chief point was to get intensity of light upon the point of operation, regardless of surrounding conditions.

*Dr. Meeker.*—Mr. President, I use both north and south lights, but I like the north best. I think that is the easiest on my eyes. It is only within a year or so that I have used glasses, and I find that I have to use them after working by a south light. I did use a stomatoscope about one month. I sold it after the second month was over, and I shall never again do any work at night. I fear I hurt my eyes by trying to do it.

*Dr. Adelberg.*—Mr. President, this is a sort of experience-meeting. I am somewhat over forty-five years of age, and I have never used a north or a south light in my office-work. For some six years I used a west light, and for nearly eight years an east light. So far I have no occasion to use glasses. It may be because I am ambidextrous, and can work at both sides of my chair. Dr. Sutphen told us that continued looking to one side was injurious to the eyes; therefore working alternately on each side of the chair may be conducive to keeping the sight good.

*Dr. Luckey.*—Mr. President, I simply want to add my voice of warning to those who may be disposed to use artificial light. I have been very much interested in the experiences of the gentlemen as they have given them forth, and I was surprised by the statement of the essayist that oculists, who work very much with artificial light, have not noticed any defects of their eyesight. Every dentist who has spoken to-night, without exception, I think, has admitted that artificial light was injurious. Why it is so I am not well enough versed in physics to say. Whether it is because the rays of light in the artificial light are not the same as they are in the natural light, I do not know, but I think probably that it is an explanation; that a clear natural light, containing all the rays of the speculum, is not so tiresome to the eye as an artificial light. But this I know from my experience, and also from the testimony of others, that one hour of work with artificial light is more straining to the eyes, and causes greater weariness and exhaustion, than two hours of work with a natural light. I was induced to buy a stomatoscope some years ago, and I tried it faithfully for a number of months. I found that my eyes were beginning to trouble me, and I discontinued its use. I did not sell it; I stowed it away as a memento of one of my mistakes. I want to add my voice to the voices of the others who have spoken, in warning to those who may feel disposed to enlarge their incomes or get ahead more rapidly by using an artificial light, not to do so.

*Dr. Sutphen.*—Mr. President, I have very little to say, in closing this discussion, except that the subject of the practicability of artificial light has been so well ventilated that it seems to have been brought much nearer to a final conclusion than by anything said by myself. Still, it would be better to use artificial light rather than poor daylight. I think it is a safe proposition that one should not use artificial light, unless he is obliged to, but I do not see how it can be especially injurious in itself. The long-continued use of it might injure the eyes by leading the operator to overwork.

INCIDENTS OF PRACTICE.

*Dr. Osmun.*—Mr. President, in the placing of artificial lower dentures in the mouth, I presume every one present who has had experience with them will admit that it is somewhat difficult at times to get a lower plate to remain in place. I know of no better way to present this fact to you in a concise manner than to relate a case where I accomplished the object I set out to do, and how I did it.

About two years ago a patient came to me to have an upper and lower denture fitted. I never saw a more unpromising mouth. It was as flat as flat could be. In the lower jaw there was no ridge whatever. The party had been in the hands of two or three gentlemen who are skilful dentists, and there had been made a number of plates of different kinds,—aluminum plates, electro-deposit plates, and so on; and they were all beautifully made, and accurately adjusted and occluded, so far as I was able to discover; but they would not stay in place. I took an upper and a lower impression, and laid them one side. While I was thinking about what I should do in the case, I was walking along the street one day and I saw some boys playing with suckers on the sidewalk, wet pieces of leather attached to a string; and I said to myself, if I could get a flexible surface to those plates, I believe they would adhere. I wrote a note to Dr. Kingsley and asked him to send me over some pieces of velum, or soft rubber. This came through a suggestion of Dr. Van Woert. I made a lower plate as we usually do, lining it with soft rubber. The adhesion of the upper plate was very defective; but the lower one which I made was perfectly firm. I then prepared a new upper plate, and lined it with velum, with a like good result. The upper one is worn in the mouth till the present day; the lower one I made over again for reasons which I will state further on.

I told my friends, Dr. Sanger and Dr. Fisher, about this case, and asked them to try this method. They will tell you their experience.

When I packed the plate, I dissolved some ordinary rubber in chloroform; coated the model, and then placed on the soft rubber. I found that this way left hard places, little particles of rubber vulcanized hard. Then some soft rubber was taken and dissolved, and used to fasten the velum on the model, and it did much better. I only covered the inside with the soft rubber, leaving a sort of ridge around the outside of the plate to finish. I found that the soft rubber left a little too much hard rubber on the outside, that cut down into the soft tissues. The lower plate was made over again, lining the entire under surface. I packed it first with a piece of blotting-paper, in order to get the thickness; then packed with rubber as we usually do. The finishing was difficult. It was rough, and uncomfortable to be worn. A disk was placed in the dental engine, and revolved rapidly, trimming down the plate until perfectly smooth.

The plate is not as clean as a hard-rubber plate, and it is only in those cases where it is exceedingly difficult to retain a lower plate that I would recommend its use. You must impress upon the patient the necessity of keeping the plate clean. And it is not as durable as a hard-rubber plate. But I think we are justified in using such plates in mouths of persons unable to wear anything else. This patient had had no comfort for some years. I have used this style of plate in perhaps half a dozen cases, and in every case it has been worn with satisfaction.

In upper dentures you sometimes find that you can retain them in this way. Try this method of lining the plate when nothing else will do. Sometimes in partial cases you find the rubber plate will rub the teeth; and in such cases if you place a little velum or soft rubber around the teeth on the plate, it will act as a spring or sponge and hold it very nicely. There are many ways that will be suggested to you as you use it. The plate has to be made a little thicker when the soft-rubber lining is used, to compensate for loss of strength.

*Dr. Sanger.*—I tried the plan of Dr. Osmun in regard to the use of velum. Dr. Kingsley kindly favored me with some soft rubber, and also gave me his advice. He said the soft rubber had been tried before; but I found, on further inquiry, that the process used, as I understand it, was to line the edges of the plate with soft rubber, forming a rim of velum and leaving the centre hard. After learning of Dr. Osmun's experiments, I made a plate for a patient in a case where I had great difficulty, making the entire surface of soft rubber. She has worn that ever since with a great deal of comfort. I judge it is over a year since I made it. She was in to see me about two months ago, and it then looked perfectly good. I can recommend this kind of plate to the gentlemen present who have not tried it. In making it you want to be careful to fasten the soft rubber down to the model, so that it will not let any portion of the hard rubber through, because if that occurs it will irritate the mouth severely. I advised my patient before I made the plate that it was an experiment, and she took it with that understanding. She has been very much pleased with the result. Probably Dr. Kingsley could give us some valuable details in regard to the working of soft rubber in this way.

*Dr. Osmun.*—In regard to fastening the soft rubber down, I have abandoned that method, and packed something else in first,—as blotting-paper,—then put on my soft rubber.

*Dr. Fish.*—Mr. President, about a year and a half ago I was struggling very hard with an exceedingly flat lower ridge, when one evening I happened to meet Dr. Osmun, and he told me about this soft rubber. I then proceeded to take my impression, and followed his instructions, with the exception that I used a soft rubber solution in chloroform to fasten the soft rubber to the plate, taking care not to have any surplus of hard rubber to crowd this out of place. After that plate was inserted in the mouth I could not dislodge it by pulling it directly upward. The plate gave the greatest satisfaction, and has, to the best of my knowledge, continued so. The mouth was exceedingly tender, and the ridge was completely absorbed. The lady had had nearly a dozen plates made in different parts of the country. I have made since that some half a dozen, and all of them are giving the best satisfaction.

In regard to soft rubber for partial dentures, I had a case that illustrated how it could be used to advantage. You know that we sometimes have patients where two or three teeth are standing at an inclination of forty-five degrees, and the plate has to be cut away in order to get it down on the ridge, and when it is down it is constantly moving. I had a case where the two canines were the only teeth remaining in the jaw, and they were inclined towards each other at an angle of about forty-five degrees, so that when the plate was cut away enough to come down on the ridge it was very difficult to keep in place. I simply filled in a little soft rubber and vulcanized it, and that plate has been worn a little over a year without trouble, and is as tight as it was when it was put in. This soft rubber is not as lasting as hard rubber, yet if it wears for a year or a year and a half without showing any depreciation, we may expect it to wear three or four years. I am using at the present time, and find my best success with, Dougherty's soft rubber.

*Dr. Richards.*—I had a case similar to Dr. Osmun's, with very flat, hard ridge. The patient had had two cases made, and they fitted very well for hard-rubber plates; but it was very difficult for her to masticate her food. I made a plate of this kind, which overcame the difficulty, and I have used it four or five times with perfect success. I had great difficulty at first in learning how to pack it, but I think I have overcome this now.

*Dr. Kingsley.*—Mr. President, unless these gentlemen insist upon the term "velum rubber," I would ask the stenographer to substitute the words soft rubber. It is not velum rubber simply, because some rubber has been used to make artificial vela.

I am not familiar with Dougherty's rubber; I presume there is color in it.

*A voice.*—It is red.

*Dr. Kingsley.*—It is objectionable. Rubber that has color in it is objectionable, and the more color there is in it the worse it is for the purpose. The very best rubber for elastic purposes that can be made contains about five per cent. of sulphur and ninety-five per cent. of the purest and best Para rubber that can be obtained. If you want to turn the color a little from black, you can add a little vermilion, not more than five per cent. In this Doughterty's rubber I should judge there was as much as twenty per cent. of coloring-matter added to it, to give it its bright color. The manufacturer evidently thinks that those that use the rubber want it colored; color means more foreign matter, and the less durable is the work. There is no special object in having a particular color; the patient does not care anything about that.

I was wondering how these gentlemen were using the soft rubber, until I inferred, from what was said, that they were vulcanizing it, in connection with hard rubber, at 320° of heat, or thereabout.

*Dr. Osmun.*—I use it both ways. I vulcanize at from 200° to 300°.

*Dr. Kingsley.*—My associate yesterday let his heater run up to 280° or 290°, in vulcanizing some soft rubber, and I told him he had spoiled the work. The best results are obtained with soft rubber by not heating above 260°; and it wants to be vulcanized from four to five hours, beginning at 240°, and running slowly up to 260°, and allowing it to remain two hours at 260°. In that way you get it very satisfactory. You cannot vulcanize hard rubber and soft rubber together at the same time and get the best results. The heat may be run up to 300°, but the work will be far less durable, will not last as long, and will be less elastic; nevertheless, it is very much better than a hard surface. The trouble that you had in trimming it up and getting a smooth surface was because of lack of experience. To get the best surface, it should be vulcanized on metal. The making of lower teeth is a little more work, I grant, but it is better. Have your cast made of tin or pipe-metal, and have it polished smooth with pumice-stone, and then vulcanize on that, and you will have a beautiful surface, as fine and smooth as glass. If the edges spread out, or if you have feather edges, take your scissors and trim the edge off to the shape you want; then take a hot instrument, and run it over the edge. That will leave a partly-melted surface, which is bad, but that is remedied by taking a rag, dipping it in chloroform, and rubbing it over the surface.

There are circumstances in which it would be inconvenient to vulcanize the hard rubber at 320°, and then subsequently attach the soft rubber and run the heat up to only 260°, but in these you can vulcanize as you have done. It will not absolutely spoil the soft rubber to run the heat up to 320°, but it injures it. If it were not for the hard rubber, it would be rotten and worthless, and it would be an imposition to give it to a patient.

*Dr. Watkins.*—I would like to ask Dr. Kingsley if he could not vulcanize soft rubber as well on a plaster model, by covering it with No. 60 tin-foil, as on a metal die.

*Dr. Kingsley.*—Yes; if the tin will hold its smooth metallic surface so that the thing will peel off from it.

*Dr. Watkins.*—I can vulcanize that way constantly hard rubber, and the tin retains its polished surface, so I do not have to polish the plate.

The subject was then passed.

